# GM-1111 reduces radiation-induced oral mucositis in mice by targeting pattern recognition receptor-mediated inflammatory signaling

**DOI:** 10.1371/journal.pone.0249343

**Published:** 2021-03-26

**Authors:** Abigail Pulsipher, Justin R. Savage, Thomas P. Kennedy, Kavita Gupta, Benjamin G. Cuiffo, Stephen T. Sonis, Won Yong Lee

**Affiliations:** 1 GlycoMira Therapeutics, Salt Lake City, Utah, United States of America; 2 Biomodels, Waltham, Massachusetts, United States of America; Northwestern University Feinberg School of Medicine, UNITED STATES

## Abstract

**Purpose:**

Oral mucositis (OM) is a common, painful side effect of radiation therapy used for the treatment of head and neck cancer (HNC). Activation of the innate immune system upon irradiation has been identified as a key precipitating event of OM. To better understand OM’s pathogenesis, we studied pattern recognition receptors (PRRs) and their downstream pro-inflammatory cytokines in a mouse model of radiation-induced OM. We also tested therapeutic efficacy of GM-1111 that targets innate immune system to reduce radiation-induced OM.

**Methods and materials:**

The pathogenesis of OM was studied in a single X-ray induced mouse model. The severity of OM was measured by visual and microscopical examinations. The irradiation-induced changes of PRRs and their downstream effector cytokine gene expression levels were determined. The efficacy of GM-1111 to reduce OM was tested in single and fractionated irradiation mouse models. The impact of the drug on tumor response to radiation therapy was also tested in a mouse model of human HNC.

**Results:**

Radiation-induced tissue ulcerations were radiation-dosage and -time dependent. The lesions showed selective increases in PRR and pro-inflammatory cytokine gene expression levels. Once daily administration of GM-1111 (≥30 mg/kg, *s*.*c*.) significantly reduced the severity and the incidence of OM. The drug had little effect on PRRs but significantly inhibited downstream pro-inflammatory cytokine genes. GM-1111 did not interfere radiation therapy to induce HNC SCC-25 tumor regression. Instead, we observed significant drug-induced tumor regression.

**Conclusions:**

Radiation induces tissue damages. The increased expression levels of PRRs and their downstream pro-inflammatory cytokine genes in the damaged tissues suggest their important contribution to the pathogenesis of OM. Drug GM-1111 that targets these innate immune molecules may be a potential drug candidate as an intervention for OM.

## Introduction

Oral mucositis is a common, painful, and potentially treatment disrupting side effect of radiation therapy used for the treatment of HNCs. Aside from its clinical toll, ulcerative OM incurs additional use of healthcare resources resulting in a significant incremental cost [1]. Despite its ubiquity amongst patients being treated with radiation for HNCs, there are no approved interventional therapeutics for OM.

Studies of OM’s pathogenesis suggest that it is the consequence of a biological cascade which continues to be defined. Nonetheless, it is clear that the initiation of OM is triggered by two key events, oxidative stress and activation of the innate immune response [2]. Pre-clinical and clinical data from studies in which interventional strategies aimed at interfering with either initiating elements suggest their value as druggable targets [3–5] and indicate that blocking initiation favorably disrupts the downstream progression of the biological events that amplify tissue injuries. In addition to its role in the initiation of OM, molecules released during cell death serve to exacerbate radiation-induced damage by continuously activating innate immune signaling pathways. These molecules are known as damage-associated molecular patterns (DAMPs, CRAMPs, or alarmins) [6–8]. Nonetheless, the sequence by which activation of the innate immune system is implicated in radiation-induced tissue injury has not been well described.

Few studies have reported potential contributions of DAMPs on pattern recognition receptors (PRRs) such as toll-like receptor (TLR) mediated pro-inflammatory signaling in radiation injuries. Canonical pathways of TLR activation induces NF-κB mediated transcriptional activation of pro-inflammatory cytokines and chemokines such as TNFα and IL-1β [2,9]. Release of these molecules recruit blood leukocytes into the site of injury and the secretion of leukocytic proteases destroys tissue matrix. The resulting increase of DAMPs in the lesion amplifies pro-inflammatory signaling that wreak havoc on the tissue [2,10]. While the potential roles of DAMPs/TLRs-mediated signaling are compelling, few studies have been reported that investigate the expression of TLRs and its downstream signaling in tissue exposed to chemoradiation. Increased expression of TLR2 and TLR4 has been reported in the THP-1 monocytes exposed to 5 Gy of X-rays [11]. More recently, Mukanyangezi *et al*. reported a 2-3-fold increased expression of TLR5 in the rat cervix exposed to 20 Gy of X-rays [12]. Nevertheless, it remains largely unknown how TLRs contribute to the tissues exposed to ionizing radiation.

Glycosaminoglycans such as hyaluronic acid and heparin are ubiquitously present in tissues and function in various physiological processes [13]. Studies suggest the potential of chemically modified glycosaminoglycans as therapeutics for inflammatory diseases [14]. GM-1111 is a synthetic glycosaminoglycan molecule (S1 Fig) with anti-inflammatory effects by inhibiting innate immune molecules [15,16]. Given the importance of innate immune system activation in the initiation of OM, we elected to test the efficacy of GM-1111 in mitigating radiation-induced OM using murine models. We further investigated the expression levels of TLRs and their downstream effector molecules in irradiation damaged lesions. To assure that such an approach would not impair the anti-tumor effects of radiation, we also studied the effects of GM-1111 on established tumor growth and on the ability of radiation therapy to induce tumor regression in an orthotopic human HNC xenograft model.

## Materials and methods

### Animals and animal husbandry

Approximately 7–8 weeks old BDF1 (Charles River Laboratories, MA) and NCr-nude mice (Taconic Biosciences; Renssalaer, NY) were used for OM and tumor xenograft studies, respectively. These animals were housed in a controlled environment (temperature, humidity, and light/dark cycle). Standard lab chow, soft feed, and water were freely accessible. The experimental protocols for oral mucositis studies (16–07008) and orthotopic tumor xenograft model studies (18-0619-1) were approved by the University of Utah and by the Biomodels’ Institutional Animal Care and Use Committee (IACUC), respectively. All experiments using laboratory animals were conducted according to the IACUC guidelines.

Animals were weighed and monitored for general health daily over the course of the study. In addition to standard diet, animals were provided with highly palatable soft food. Supportive care in the form of sterile saline (*s*.*c*.) was administered if an animal lost >15% of its initial body weight. Animals with severe body weight loss (> 20% for oral mucositis study; > 30% for tumor xenograft study), unable to gain food/water, be in pain, distress, or moribund were euthanized for humane reasons.

### Effects of GM-1111 on TLR2-activation and pyroptosis

HEK-Blue^™^ hTLR2 (#hkb-htlr2, InvivoGen, CA) and THP1-HMGB1-Lucia^™^ (#thp-gb1lc, InvivoGen) cells were maintained and the experiments were conducted according to the supplier’s instructions. HEK-Blue hTLR2 cells were maintained in DMEM supplemented with 50 U/mL penicillin, 50 μg/mL streptomycin, 10% heat-inactivated fetal bovine serum (FBSi), and HEK-Blue^™^ Selection reagent (InvivoGen) in 75-cm^2^ flasks. THP1-HMGB1-Lucia cells were maintained in RPMI 1640 medium supplemented with 2 mM L-Glutamine, 25 mM HEPES, 10% FBSi, 100 U/mL penicillin, 100 μg/mL streptomycin, and 100 μg/mL Normocin.

hTLR2 cells stably express human TLR2 along with a reporter protein (secreted alkaline phosphatase, SEAP), which is under the transcriptional control by NF-κB and AP-1. Cultured hTLR2 cells were harvested with PBS. After incubating cells with drug for 30 min, TLR2 was activated with its specific agonist Pam3CSK4 (#tlrl-pms, InvivoGen) overnight at 37°C. Aliquots of culture medium were tested for TLR2 activation by measuring SEAP activity using the Quanti-Blue assay reagent (#rep-qbs, InvivoGen).

THP-1-HMGB1-Lucia cells secrete recombinant HMGB1::Lucia luciferase fusion protein when the cells undergo pyroptosis. THP1 cells grown in culture flasks were collected in PBS and incubated with drug for 30 min. Pyroptosis was induced with successive stimulation with *Escherichia coli* LPS (#tlrl-b5lps, InvivoGen) and nigericin overnight. The quantity of HMGB1::Lucia in the cell culture medium was determined by measuring the luminescence with Quanti-Luc (#rep-qlc1, Invivogen) reagent.

### X-ray irradiation induced oral mucositis

For head-only irradiation, the body of the animals were shielded with lead blocks while exposing the head. X-rays were generated by RS2000 Biological Research Irradiator (Rad Source Technologies, GA) with a dosage rate of 1.9 Gy/min with instrument parameters set to 160 kV/25 mA. The radiation dosage was monitored with Radcal^®^ Accu-Dose dosimeter (Model 2086, CA). GM-1111 was dissolved in sterile PBS and dosed once daily. On day 8 (single irradiation) or day 9 (fractionated irradiation), all animals were euthanized and the tongues were harvested. The tongue samples were then stained with Toluidine Blue to visualize the ulcerative lesions [17], photographed, and then fixed in formalin for histological examination.

### Gene expression analysis

Expressed genes in the mouse tongue tissues embedded in paraffin were determined using the nCounter System and Mouse Inflammation V2 Panels (nanoString Technologies, WA). Extracted nucleic acid samples were hybridized overnight at 65°C in a mixture of total RNA samples, nCounter reporter probes, and nCounter capture probes. The target genes were quantified with the nCounter Digital Analyzer/nSolver platform. Six housekeeping genes (*Cltc*, *Gapdh*, *Gusb*, *Hprt*, *Pgk1*, and *Tubb5*) were used for background subtraction and normalization of the raw mRNA transcript copy counts.

### Human HNC orthotopic xenograft study

A human tongue squamous cell carcinoma cell line SCC-25 (ATCC #CRL-1628) was engineered to constitutively express Luc2 luciferase reporter gene and cultured in a 1:1 mixture of DMEM and Ham’s F12 medium supplemented with 400ng/mL hydrocortisone, 10% FBS and penicillin/streptomycin. On the day of xenograft, SCC-25-Luc cells were suspended in a serum-free medium and inoculated (10^6^ cells/30 μL) into the rostral tongue in isoflurane (2%) anesthetized animals. Tumor growth was monitored twice per week with whole-body bioluminescence imaging using Lumina Series III In-Vivo Imaging System (PerkinElmer). On the day of imaging, animals were administered with 150mg/kg (*i*.*p*.) D-luciferin as a substrate for luciferase and imaged 15–20 min later. Tumor volumes were expressed as total radiance flux (TRF; photons/second or ph/s).

Animals in Group 1 were treated with vehicle (PBS) or GM-1111 (30 mg/kg), once daily (*s*.*c*.) from the day of tumor cell inoculation for 19 days. Animals in Group 2 were randomized 20 days after tumor cell inoculation into four treatment groups with similar mean TRF (8.67 x 10^7^ ph/s). On day 20, two arms in Group 2 were anesthetized with xylazine (5 mg/kg)/ketamine (100 mg/kg) and irradiated once with X-rays (30 Gy, 1 Gy/min) which were targeted to the tumor in the tongue. X-rays were generated by a 160 kVp (15-ma) X-ray source at a focal distance of 25cm, hardened with a 0.35 mm copper filtration system at a rate of 1 Gy/min. The rest of the body was covered with a lead shield while exposing the tumor. Animals were continuously monitored as they fully recovered from anesthesia (isoflurane 1.5–2%) on a heated pad, and then immediately returned to their home cage. At the end of the experiment, all animals were euthanized with CO_2_.

### Histological examinations

Formalin-fixed tongue tissues were processed for paraffin embedding and the embedded tissues were sectioned at 4 μm thickness and stained with hematoxylin and eosin (H&E). Histological observations were graded for the severity of tissue damage in each category (Table 1) and the sum of these grades were used to designate the severity of each sample. Scoring was made in a blind fashion by obscuring the group identity of the samples to the examiner.

**Table 1:** Microscopical assessment criteria for histological score determination.

Tissues	Region	Severity (scores)
Keratin Layer (Loss)	Caudo-dorsal half	none (0), up to 1/4 (1), up to 1/2 (2)
	Rest of the tongue	normal (0), thin (1), absent (2)
Epithelium (Loss)	Near the base (dorsal surface)	normal (0), thin (1), absent (2)
Lamina Propria (Thickness)	Near the base (dorsal surface)	normal (0), slightly enlarged (1), notable (2)
Polymorphonuclear Leukocytes	Near the base (dorsal surface)	absent (0), a few cells (1), plenty (2)
Salivary glands (Glandular content)	Serous Gland	normal (0), slightly reduced (1), nearly empty (2)
	Mucous Gland	normal (0), slightly reduced (1), nearly empty (2)

### Statistical analysis

All measurement data were tested for homogeneity with either Bartlett’s test or Fligner Killeen test for equal variances as well as visual inspection of histograms to check the distribution of data. Gene expression data were first normalized, and the ratio of difference in the means of the log-transformed normalized data to the square root of the sum of the variances of samples. The mean differences among treatment groups were tested for statistical significance by *one-way* analysis of variance test followed by Dunnett’s *t*-test or Tukey’s multiple comparison test as *post hoc*. Histological severity scores were analyzed with Kruskal-Wallis test followed by nonparametric multiple comparison test procedure using the *nparcomp* package [18]. Statistical analyses were done with R statistical analysis software (Version 3.6.2). Curve fittings for TLR2 and pyroptosis inhibition data were done with SciDAVis (Version 1.2.6).

## Results

### Ionizing radiation induces extensive ulcerative lesions in the tongue

We investigated the pathogenesis of radiation-induced OM in mice. Specifically, we studied the development of lingual lesions as a measure of OM because the ease of studying tongue tissues [19,20]. To delineate the progress of the lingual lesions, we irradiated the head of the mouse with a single dose of X-rays at 15 Gy and 20 Gy. On day 5 and 8 post-irradiation, the mouse tongues were harvested and the ulcerative lesions were visualized with Toluidine Blue [19–21].

**Fig 1:**
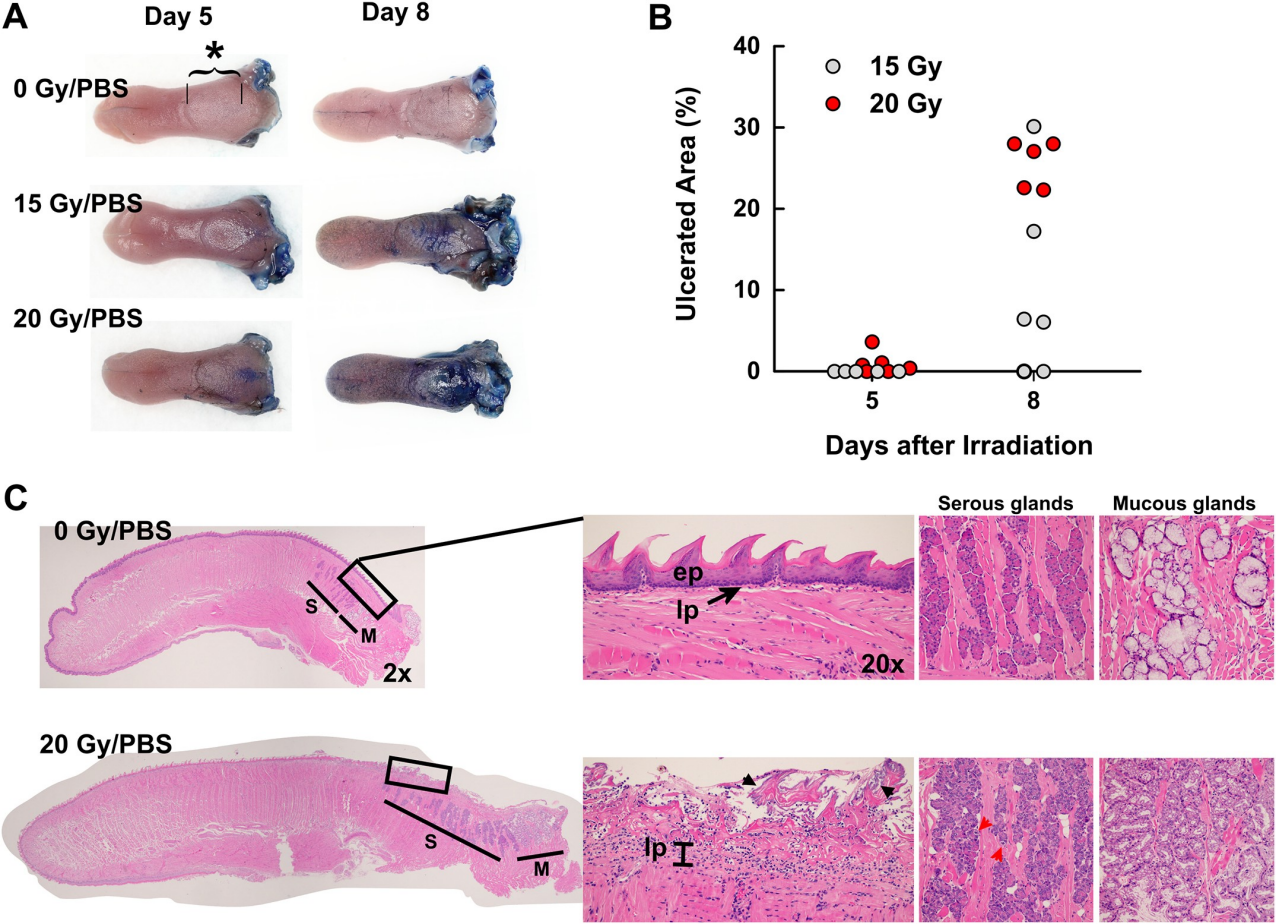
Progress of radiation-induced oral mucositis in mice. **A**, Ulcerations primarily occurred around the intermolar eminence (*, bracketed area). **B**, Measurements of ulcerated areas. **C**, Photomicrographs of the tongues (H&E stain). Arrow heads point to colonized microbes. **ep**, epithelium, **lp**, lamina propria.

As shown in Fig 1A, the ulcerative lesions mostly developed in the dorsal surface of the base—primarily in the intermolar eminence. Anatomically, the epithelium in this region is made of a few cell layers that gradually become thinner towards the pharynx and are covered with thin keratin substance. Morphologically, the epithelium in the base of the tongue is similar to the buccal mucosa in humans and suggests a potential surrogate measure of general oral mucosal changes. Lesions were apparent 8 days post-irradiation in the 20 Gy group but less apparent in the 15 Gy group suggesting the severity of the lesions appeared to be radiation-dosage dependent (Fig 1B).

Microscopically, the entire surface of the tongues showed radiation dosage- and time-dependent epithelial destruction. However, the most notable changes occurred near the intermolar eminence (Fig 1C). The rostral portion of the tongue also showed dramatic epithelial cell loss and the remaining cells appeared with a mixture of necrotic and non-necrotic morphological changes. The keratin layer at the base of the tongue had mostly disappeared by day 8, and the bared lesions were usually colonized by microbes (Fig 1C, arrowheads). Numerous polymorphonuclear leukocytes (PMNs) throughout the surface and lamina propria (**lp**). These PMNs were also present near the microbes and dead tissues (S2 Fig).

We also observed morphological alterations in the salivary glands located near the base of the tongue [22]. Most notably, glandular contents were markedly reduced with the resulting large and round nuclei of the acinar cells that normally present as thin spindle shapes due to the pressure of the glandular materials (Fig 1C). Reduced salivary glandular contents in the tongue suggest a potentially reduced saliva needed for proper lubrication and digestion. By contrast, the muscles in the tongue were mostly unaffected by the radiation dosage range and observational time points. Overall, irradiation-damaged tissues showed progressive degenerative to ulcerative lesions with the infiltration of a large number of inflammatory leukocytes and the colonization of microbes. Alterations in the salivary glands suggest potentially dysfunctional glands and potential reduction of saliva on the tongue.

### Ionizing radiation increases pro-inflammatory gene expression

**Fig 2:**
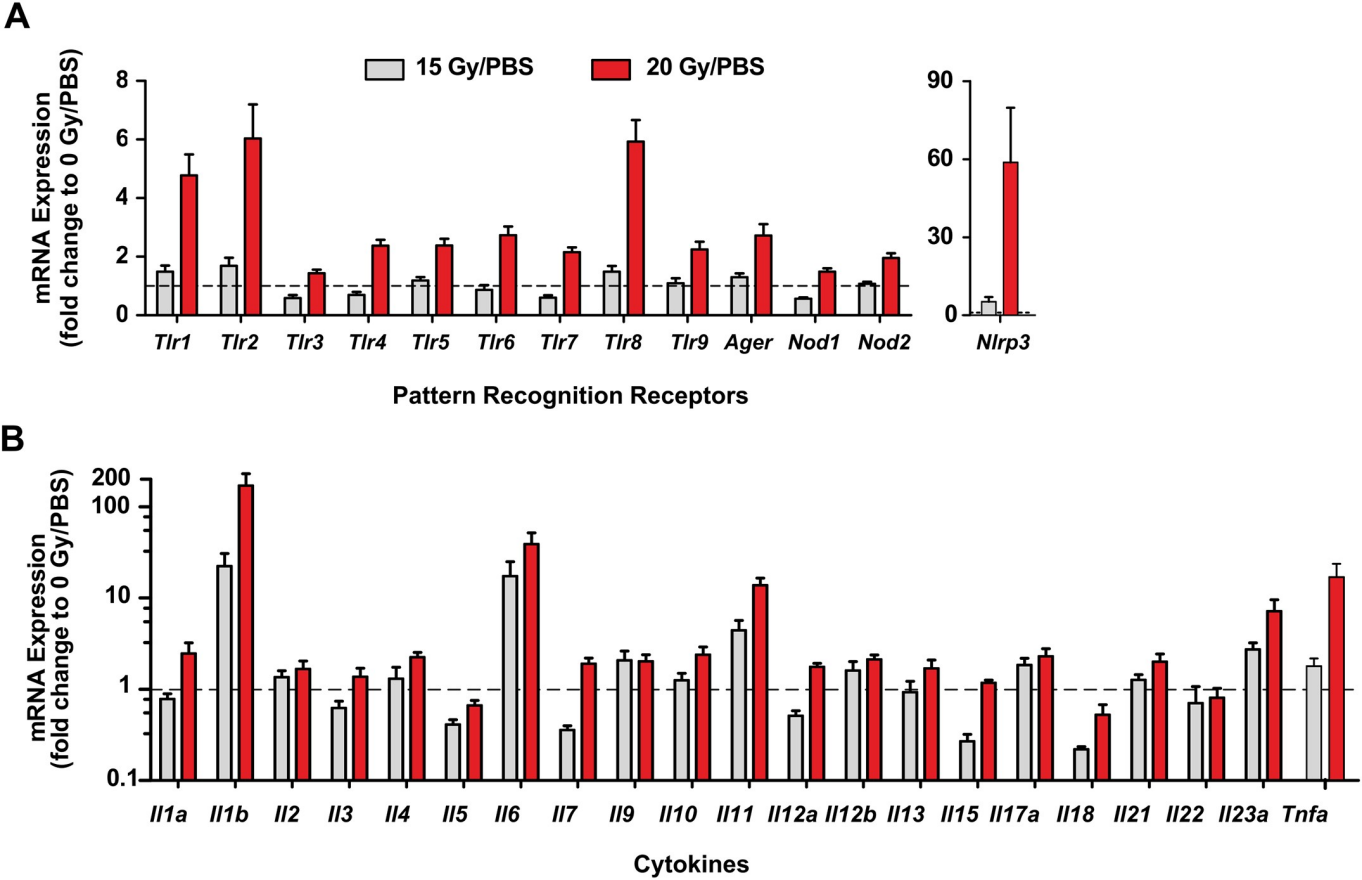
Altered gene expression in radiation-damaged lesions. Multiplex gene expression analyses of PRRs (**A**) and cytokines (**B**, log scale) in the tongue tissues (day 8 post-irradiation). Bars represent the mean values and the error bars are SEM (n = 6 for 15 Gy/PBS; n = 9 for 20 Gy/PBS).

To elucidate the molecular underpinnings that drive radiation-induced tissue damage, we determined the changes of genes involved in inflammation: PRRs and their cellular downstream signaling cytokines. Two mm diameter tissue samples (paraffin embedded tissues) from the dorsal surface in the intermolar eminence were used for the gene expression analyses (Fig 1C, boxed regions). This region was chosen for the analysis because it shows the most severe radiation-induced inflammatory changes. No apparent changes in gene expression levels were observed in the 5-day post-irradiation tissue samples in the 15 and 20 Gy irradiation groups (S3 and S4 Figs). Also, PRR genes in the tissues from 8-day post-irradiation did not show apparent changes in the 15 Gy irradiation group (Fig 2A). By contrast, the tissue samples from the 20 Gy irradiation group showed rather selective changes in expression levels of PRR genes. For example, the expression levels of *Tlr1*, *Tlr2*, *Tlr8*, and *Nlrp3 in the irradiated animals* were increased over 5-fold compared to controls while *Tlr4-7*, *Tlr9*, and *Ager* were elevated by about 2-fold. On the cell surface, TLR2 functions as a heterodimer with TLR1 or TLR6. The increased expression of *Tlr2/1* gene suggest that TLR2 likely contributed to the development of the inflammatory lesions. Unlike other TLRs, the physiological significance of TLR8 in mice is largely unknown. A nearly 60-fold increase in *Nlrp3* expression was noted in the 20 Gy irradiation group. Because NLRP3 inflammasome contributes to pyroptotic cell death, the increased expression of *Nlrp3* in the radiation-damaged tissues suggests that the NLRP3 inflammasome may contribute substantially to the development of the inflammatory lesions.

Unlike the modest changes in PRR gene expression levels, the expression of certain cytokine genes was substantially increased in the 15 Gy irradiation group. These include *Il1b*, *Il6*, *Il11*, and *Il23* with over 20-fold increases compared to controls (Fig 1B). The expression levels of these cytokines along with *Tnfa* were further increased in the 20 Gy irradiation group. Activated as downstream cell signaling of TLRs/NLRP3 inflammasome, these cytokines contribute to the initiation of inflammatory changes in the tissue. Overall, these data suggest that PRR-mediated signaling may play important roles in the development of radiation-induced lesions.

### GM-1111 blocks TLR-mediated cell signaling and pyroptosis

**Fig 3:**
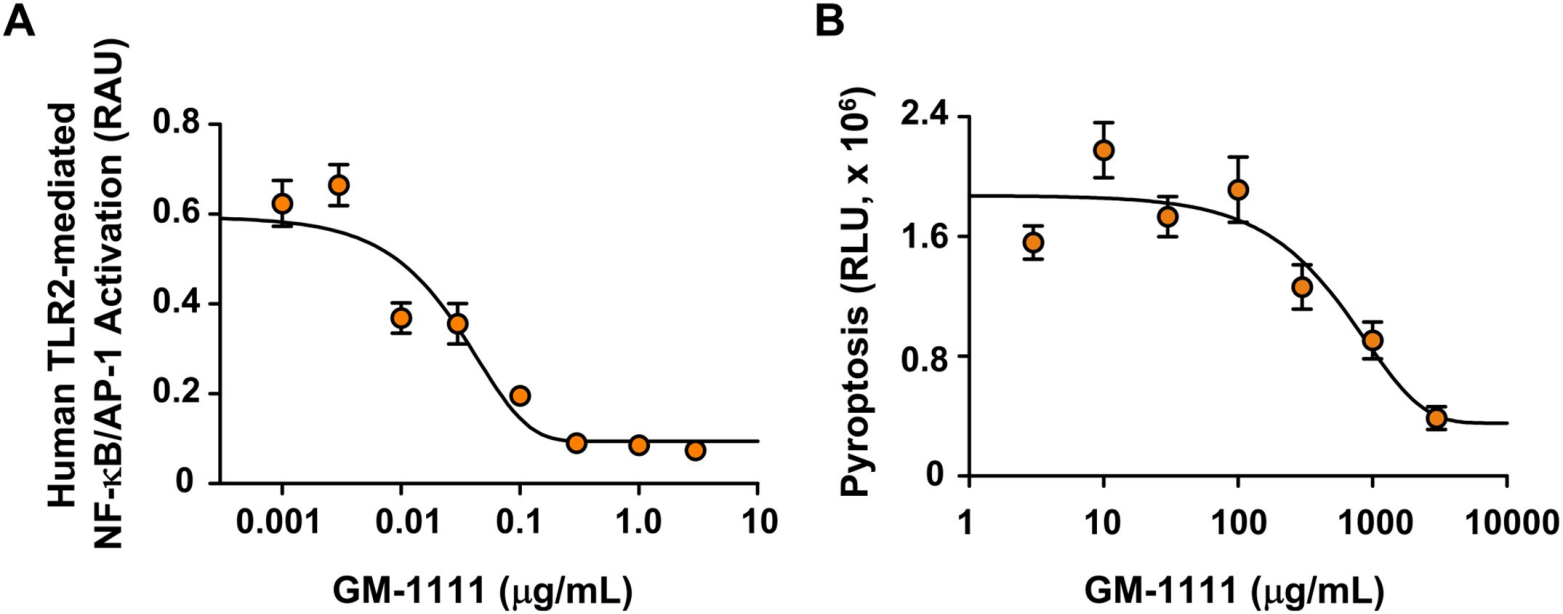
GM-1111 inhibits TLR2-induced pro-inflammatory cytokine signaling and NLRP3 inflammasome mediated pyroptosis. **A**, Human TLR2 was activated with TLR2-specific agonist Pam3CSK4 (3 ng/mL) in the presence of variable concentrations of GM-1111. **B**, NLRP3 inflammasome mediated pyroptosis was tested in human THP1 cells. Symbols represent the mean values and the error bars are SEM (n = 5 in **A**; n = 8 in **B** per concentration).

The effects of GM-1111 on TLR2-mediated cell signaling as well as NLRP3 inflammasome mediated pyroptosis were tested *in vitro* using two specifically engineered cell lines (HEK-Blue hTLR2 and THPR1-HMGB1-Lucia cells), which are designed to verify specific inhibitors and agonists. First, the inhibitory effects of GM-1111 on TLR2-mediated pro-inflammatory cell activation were determined by incubating hTLR2 cells with variable concentrations of GM-1111 and then stimulated with TLR2-specific agonist Pam3CSK4. Activated hTLR2 in these cells triggers NF-κB to produce and release a reporter protein (SEAP) into the cell culture medium that is measured by the enzymatic degradation of its substrate (Fig 3A). Cells incubated with GM-1111 showed a drug concentration-dependent reduction of SEAP with an IC_50_ value of 30.5 ng/mL (Fig 3A) suggesting that GM-1111 is a potent inhibitor of TLR2-mediated pro-inflammatory cell signaling.

Next, we tested the effects of GM-1111 on NLRP3 inflammasome mediated pyroptotic cell death using THP1-HMGB1-Lucia cells. These cells release HMGB1::Lucia (HMGB1 fused to Lucia luciferase) as a reporter protein into the cell culture medium when the cell membrane ruptures by the NLRP3 inflammasome activation through successive stimulation with LPS and nigericin to activate caspase-1. Cells incubated with varying concentrations of GM-1111 produced modest (IC_50_ value of 610 μg/mL) but drug dose-dependent inhibitory effects on the pyroptosis in the test system (Fig 3B). These data suggest that GM-1111 has the potential to reduce NLRP3 inflammasome activation and pyroptotic cell deaths.

### GM-1111 reduces single X-ray irradiation induced oral mucositis

**Fig 4:**
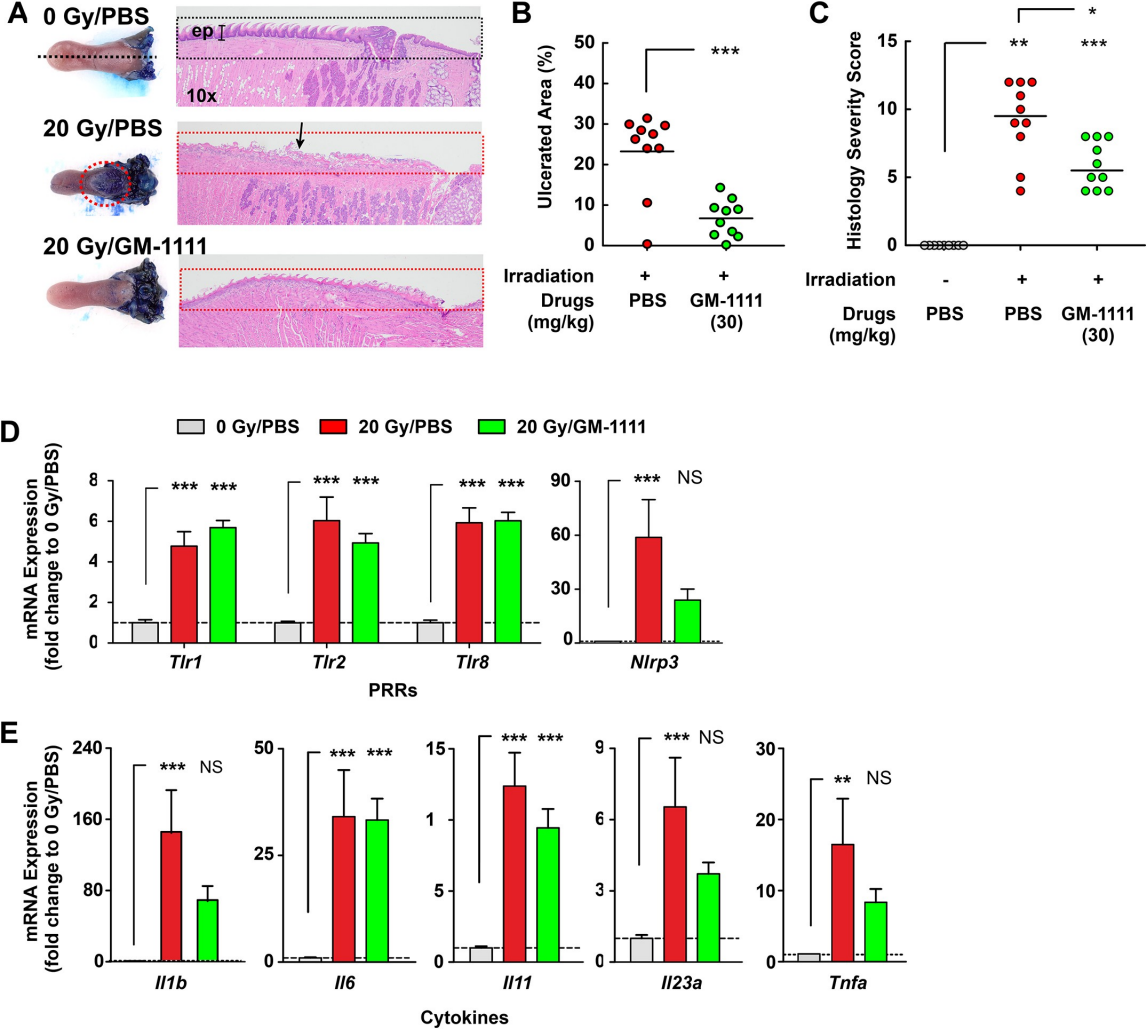
GM-1111 reduces single X-ray irradiation-induced oral mucositis. **A**, Photographs (left panel) and photomicrographs (right panel) of representative tongues for each treatment group. Measurements of ulcerative lesions (**B**) and histological severity scores (**C**). Symbols represent individual values and bars are the mean values for each group. PRR (**D**) and cytokine (**E**) gene expression levels in the lesions (n = 9 per group). ** *p* < 0.01, *** *p* < 0.001, and NS (not significant, *p* > 0.05).

The increased expression of PRRs and their downstream effector molecules in the radiation damaged epithelium suggest that targeting these pro-inflammatory signaling molecules may reduce the severity of OM. Our experimental data demonstrated that GM-1111 inhibits PRRs, suggesting its potential to reduce OM. To test this hypothesis, GM-1111 was subcutaneously administered (30 mg/kg) once daily to mice starting from 24 hrs after X-ray irradiation (day 0) to day 7. As controls, two groups of animals were dosed at the same schedule with PBS, which was used as a vehicle for GM-1111. One of these groups was irradiated with X-rays (20 Gy) to serve as a disease control and the other group of animals was not irradiated to serve as healthy control. On day 8, we harvested the tongues and examined the extent of mucosal inflammation. Fig 4A illustrates the tongues from the PBS/20 Gy irradiation group with severe ulcerative lesions, which were confirmed by microscopical examination. By contrast, GM-1111 treated animals had smaller lesions throughout the tongue with a thin but structurally intact barrier.

Subsequent measurements of the ulcerative areas showed that most (8 out of 10) of the PBS treated animals had ulcerative lesions spanning 20–30% of the dorsal surface of the tongue (Fig 4B). The lesions were significantly smaller (less than 10%) in the GM-1111 treatment group than in the PBS treated controls. Histological examinations were categorized to assess the severity of tissue damage as scores and the results were consistent to the visual assessment with substantially reduced histological severity score in GM-1111 treatment group compared to the PBS treatment group (Fig 4C). These results suggest that GM-1111 treatment can reduce radiation-induced oral tissue damage.

Next, we asked whether GM-1111 treatment ameliorated the expression levels of PRR and cytokine genes induced by irradiation. Multiplex gene expression analysis data showed that the GM-1111 treatment group did not change the radiation-induced increase of *Tlr1*, *Tlr2*, and *Tlr8* expressions (Fig 4D). However, the expression levels of *Nlrp3* in drug treated animals were significantly mitigated compared to the vehicle treatment group suggesting that GM-1111 possibly affected NLRP3 inflammasome mediated pyroptosis in the lesions. In addition, the GM-1111 treatment group showed significant mitigating effects on the expression of pro-inflammatory cytokines such as *Il1b*, *Il23a*, and *Tnfa* (Fig 4E) that were increased in the lesions (Fig 2B). However, the expression levels of *Il6* and *Il11* remained increased in the GM-1111 treatment group. Overall, these data suggest that GM-1111 treatment ameliorates the radiation-induced expression of pro-inflammatory genes that are downstream of PRRs.

### GM-1111 is effective in reducing fractionated irradiation induced oral mucositis

**Fig 5:**
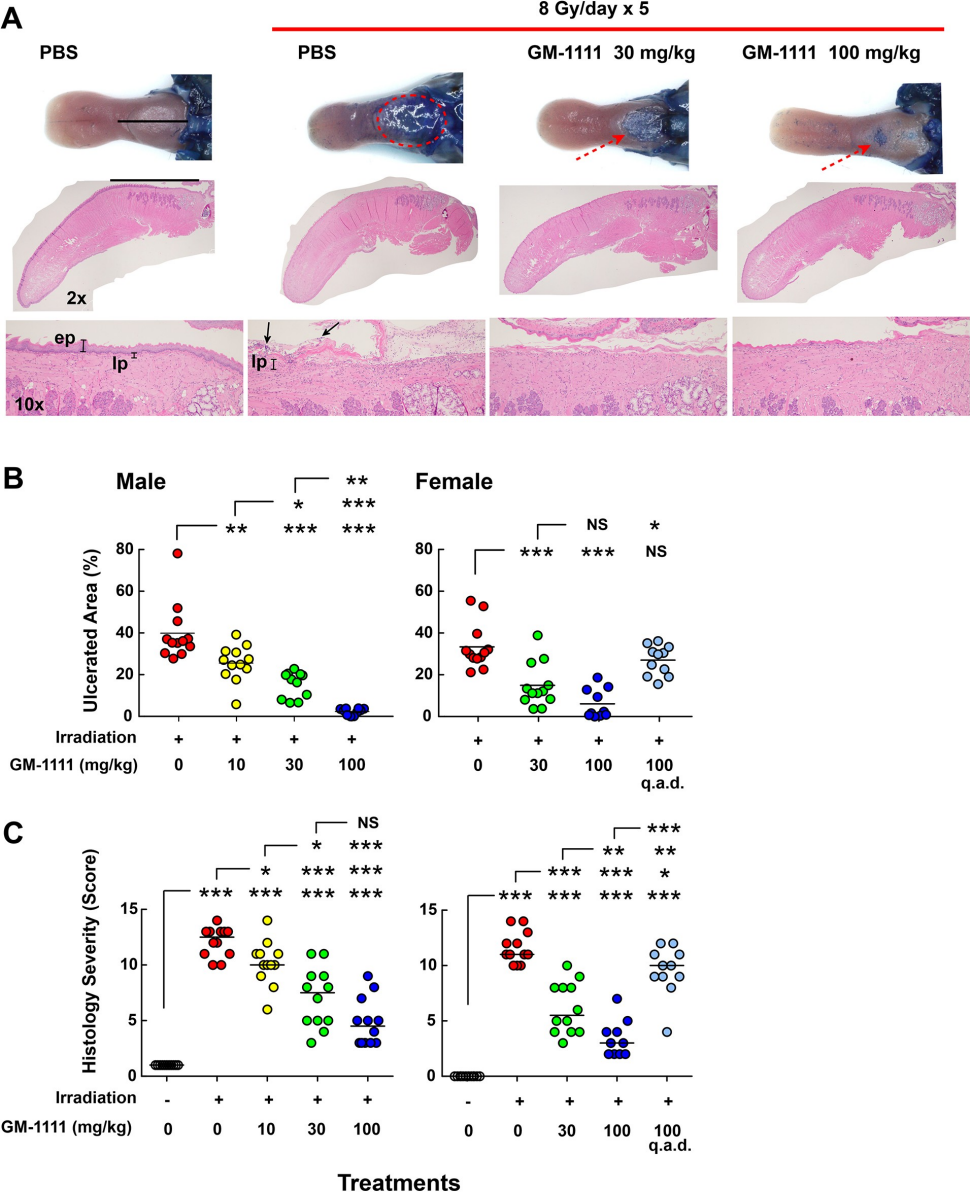
GM-1111 reduces oral mucositis induced by fractionated irradiation. **A**, Top row: tongue samples showing the ulcerative lesions (highlighted in red, stained with Toluidine blue). Middle row: photomicrographs (H&E stained) illustrating the ulcerative lesions. Higher magnification photomicrographs (bottom row) showing the lesions in the intermolar eminence. **ep**, epithelial layer; lp, lamina propria. Arrows indicate colonized microbes. Measurements of ulcerated areas (**B**) and histological severity scores (**C**). *p < 0.05, **p < 0.01, ***p < 0.001, and NS (not significant, *p* > 0.05).

Radiation therapy for HNC treatment is typically fractionated into smaller doses to minimize cutaneous injuries. Even with this kind of effort, most people undergoing external beam therapy (40 Gy or higher) experience severe OM. To generate OM with fractionated irradiation, we exposed both male and female mice with once daily 8 Gy of X-ray irradiation for 5 consecutive days (cumulative dose of 40 Gy). With this irradiation schedule, the effects of OM peaked 9 days after the first irradiation and the extent of ulceration was substantial. To verify the therapeutic effects and to find an effective dose range of GM-1111 to reduce OM, groups of animals were subcutaneously administered with once daily GM-1111 (0, 10, 30, or 100 mg/kg) from 1 day after the first irradiation until day 8. On day 9, harvested tongues were examined for lingual ulcerations and all drug dosing groups showed reduced ulcerations (Fig 5). The lesions were GM-1111 dose-dependently reduced (Fig 5B) that were consistent to our microscopical examination as revealed in severity scores (Fig 5C). In the female study group, we dosed animals with GM-1111 once daily at 30 and 100 mg/kg. Instead of testing 10 mg/kg of GM-1111 dosage that showed little to modest effects, we dosed the remaining study arm with GM-1111 at 100 mg/kg GM-1111 once every other day (q.a.d.) to investigate whether the drug can be dosed less frequently. However, this dosing regimen did not reduce the OM (Fig 5B and 5C) suggesting that once every other day GM-1111 dosing was insufficient to reduce the development of OM.

Overall, these data demonstrated that once daily GM-1111 administration was effective in reducing OM induced by fractionated irradiation. These therapeutic effects of GM-1111 were comparable in both sexes of animals.

#### GM-1111 does not interfere with tumor targeted radiation therapy

**Fig 6:**
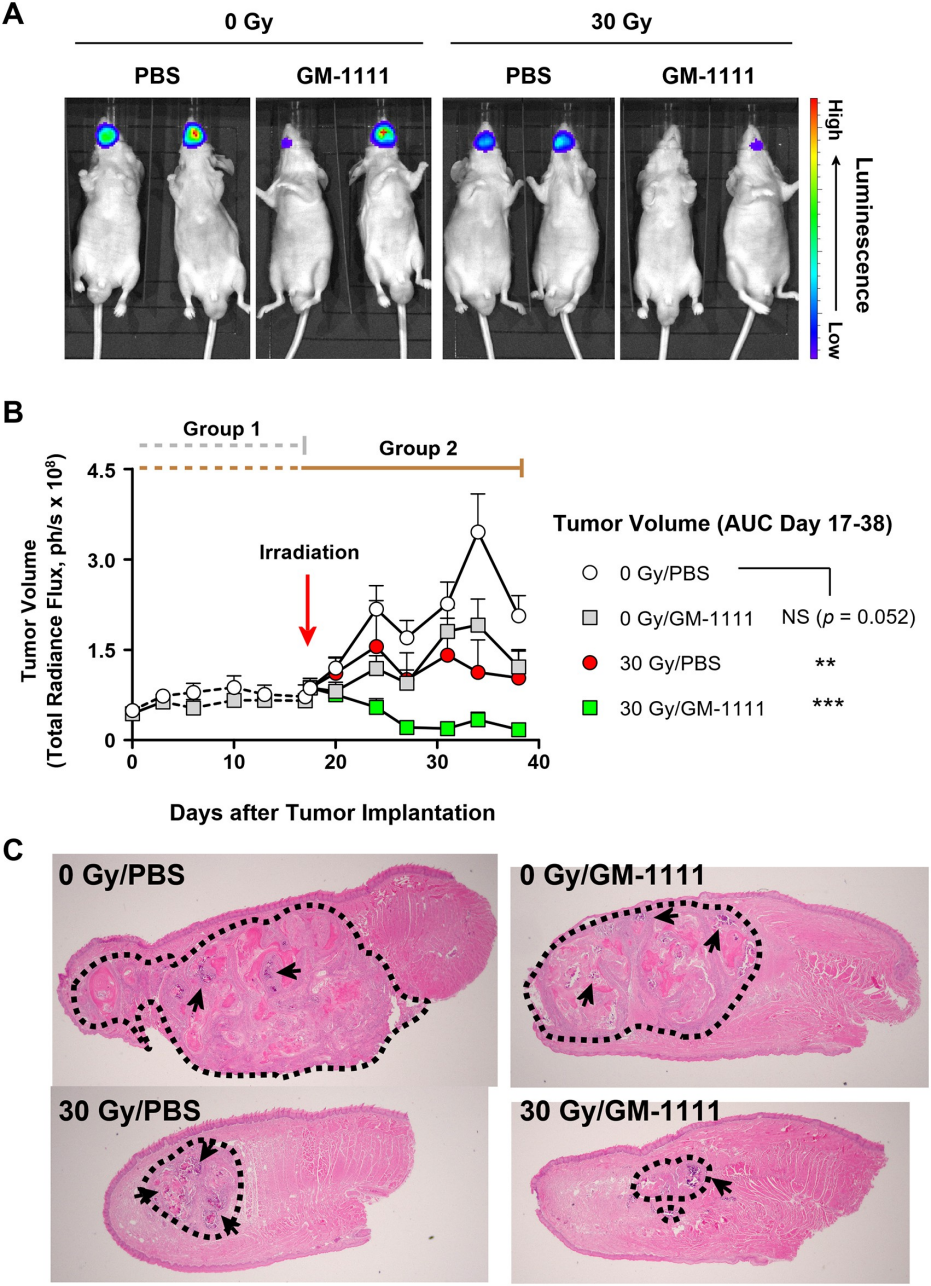
Treatment with GM-1111 does not interfere with radiation therapy. **A**, *In vivo* bioluminescence imaging of SCC-25-Luc cell tumors (day 38). **B**, Changes in tumor volume over time. Symbols are the mean values and error bars are SEM (upward direction). Area under the curve (AUC) values are calculated for Group 2 (day 17–38). **p < 0.01, ***p < 0.001, and NS (not significant, *p* > 0.05). **C**, Photomicrographs of tongues samples. Tumors are marked with dotted lines. Arrows indicate necrotic foci. Sagittal section (H&E stain, original magnification 2x).

We tested whether GM-1111’s radiation-damage mitigating effects would interfere with radiation therapy to induce tumor regression in a mouse model of orthotopic human HNC using a human tongue cancer cell line, SCC-25-Luc cells. First, we investigated whether GM-1111 had effects on the cancer cell growth as well as on tumor formation in Group 1 by starting drug dosing from the day of tumor cell implantation until day 17 when the solid tumors formed in the vehicle (PBS) treatment group. Next, Group 2 was designed to test whether GM-1111 negatively affects radiation therapy on the established tumor. Group 2 was divided into two subgroups receiving either PBS or GM-1111 in combination with 30 Gy of X-ray irradiation. In both groups 1 and 2, the implanted cancer cells formed tumors that were strictly restricted to the tongue (Fig 6A). Both PBS and GM-1111 treatment resulted in comparable tumor growth in Group 1 suggesting that GM-1111 did not affect cancer cell growth or tumor establishment (Fig 6B). The tumor growths in Group 2 were noticeably reduced in the radiation therapy groups. The radiation-induced tumor regression was evident with a significant reduction in tumor volume observed in the 30 Gy/PBS treatment group (Fig 6B). In addition, we also observed significant tumor regression in the animals administered with GM-1111. The anti-tumor effects of GM-1111 were robust when combined (30 Gy/GM-1111) with radiation therapy with the tumor volume further regressing below its initial level.

Microscopical examination demonstrated that the cancer cells were mostly encapsulated by fibrous tissues while occupying the skeletal muscle layer. Compared to the 0 Gy/PBS treatment group, the tongue samples in teh 0 Gy/GM-1111, 30 Gy/PBS, and 30 Gy/GM-1111 treatment groups had smaller tumors containing prominent necrotic foci (Fig 6C, arrows), reduced malignant cells, and less prominent fibrous tissue. Overall, these data demonstrate that subcutaneous administration of GM-1111 does not interfere with ongoing radiation therapy in SCC-25 cell tumors.

## Discussion

We show that the glycosaminoglycan-based drug GM-1111 reduces radiation-induced OM in mice. The drug targets TLR-mediated pro-inflammatory cell signaling and the NLRP3 inflammasome. Radiation damaged tissue releases various cellular and extracellular materials that function as potent inflammatory DAMPs. Bombarded by the increased flux of DAMPs, activated PRRs present in the neighboring cells produce and release of pro-inflammatory cytokines and chemokines. The inhibition of DAMPs/TLRs cell signaling by GM-1111 likely reduces the amplification of pro-inflammatory cell signaling that causes tissues into a self-destructive state. While the concept of DAMPs/TLRs activations in radiation induced tissue damage is not new [23], the contributions of these molecules to the pathogenesis of radiation-induced tissue damage are slowly emerging. The significant upregulations of cell surface TLR2 and TLR4 by ≥ 5 Gy irradiation in THP1 monocytic cells were reported [11]. Mukanyangez *et al*. reported a modest 2-fold increase in TLR5 with no changes in TLR2/TLR4 in rat cervical tissues harvested 14 days after a 20 Gy irradiation [12]. Our studies demonstrated marked increase in *Tlr1*, *Tlr2*, and *Tlr8* with modest increases in other types of TLRs. These data suggest that various types of TLRs present in the tissues may contribute to radiation-induced tissue injuries.

We observed increased expression levels of TLRs and cytokines in radiation damaged tissues. What drives the expression of these genes? The understanding of molecular mechanisms regulating the expression of innate immune molecules could give us insight into the pathogenesis of radiation induced tissue damage. Studies demonstrated that pro-inflammatory cytokines affect TLR2 gene expression through NF-κB in mouse macrophages and hepatocytes while the same cytokines did not alter TLR4 gene expression suggesting the possibility of the independent regulation of TLR genes [24,25]. A study by Shatz *et al*. demonstrating that specific stimuli regulate the expression of TLR genes support such a possibility [26]. Similarly, NLRP3 gene expression also appeared to be dependent on cytokines and NF-κB [27]. Adding complexity to the potential stimulus specific regulatory mechanism, a potential crosstalk between TLRs and inflammasomes as a regulatory mechanism of these genes has been suggested [28]. We speculate that the early DAMP-mediated release of pro-inflammatory cytokines in irradiated tissues likely induces PRR/cytokine genes in the lesions as demonstrated in our studies. The increased expression of pro-inflammatory cytokines without altering PRR genes in the 15 Gy irradiation group suggests that the induction of PRR genes requires considerable increases of their regulatory molecules that arise from high dosage radiation exposure. Consequently, increased PRRs can amplify the inflammatory changes. We speculate that this may be a strategic point of intervention to reduce tissue damage.

The contribution of NLRP3 inflammasome in inflammatory diseases has been well recognized since its discovery [29]. Our study data illustrate that radiation-damaged tissues have increased NLRP3 gene expression, and we suspect that NLRP3 inflammasome activation may be an important contributor in the pathogenesis of radiation-induced OM. Both the activation of PRRs and the production of IL-1β are critical for inflammasome activation. Ishihara *et al*. demonstrated that high dose irradiation (20 Gy) can induce *Il1b* expression in mouse splenic cells [30,31], with increased activation of NLRP3 inflammasome in the intestinal epithelium [32,33] and macrophages [34,35]. Upon inflammasome activation, cells can undergo highly inflammatory pyroptotic cell death due to the consequential release of DAMPs into the tissues [36]. Hence, targeting inflammasome activation seems like a logical strategy to minimize tissue damage [29]. Such possibilities have been suggested in the studies to counter radiation-induced enteritis with melatonin [33] and pulmonary vascular damage with LGM2605 [37]. Consistent with these studies, our data show that GM-1111 inhibits NLRP3 mediated pyroptosis, and this effect may contribute to reducing OM. Also, inhibiting TLR2 by GM-1111 would reduce the release of pro-inflammatory cytokines that facilitate inflammasome activation ultimately reducing NLRP3 inflammasome activation. We demonstrated the inhibitory effects of GM-1111 on NLRP3 inflammasome mediated pyroptosis using a human THP1 monocytic cell line. It should be noted that our experimental system relied on specific ligands to activate NLRP3 inflammasome. Instead of using cellular debris after irradiation, we used LPS as a DAMP to initiate TLR4-mediated primary signal for inflammasome activation due to the complexity of cellular materials released in the radiation-damaged tissues. TLRs and most PRRs have weak agonist selectivity. As such, we speculate that the inhibitory effect of GM-1111 on NLRP3 inflammasome mediated pyroptosis is one of the important contributing factors to reduce radiation-induced tissue damage.

Because GM-1111 is intended to reduce OM during radiation therapy, it is important to question whether the drug penetrates into tumor tissues and negatively impacts radiation therapy designed to induce tumor regression. Such concerns have been addressed with Palifermin and other experimental drugs [38,39]. Instead of protecting tumors from radiation therapy, we observed the anti-tumor effects of GM-1111 on the established tumor that were further enhanced by concomitant radiation therapy. The ability of GM-1111 to induce tumor regression without affecting cancer cell growth suggests that the drug may target the tumor microenvironment instead of the tumor cells themselves. Glycosaminoglycans have shown such potentials to affect tumor growth by modulating growth factors and chemokines [40].

In most OM patients, ulcerative lesions primarily occur in the nonkeratinized tissues such as the labial and buccal mucosa [41]. By contrast, rodent oral tissues are comprised of well keratinized epithelia [42,43]. These anatomical differences between the rodent and human oral mucosa raise the question of whether the pathogenesis of OM is similar among these species. In the mouse tongue, the mucosal tissues in the intermolar eminence have a less developed keratin layer. The predominant occurrence of ulcerative lesions in the intermolar eminence of the tongue as demonstrated in our studies and also by others [17], suggests that the keratin layer may play important roles in delaying ulcerations. Taken together, the mouse tongue appears to be a good model to assess the pathobiology of OM. The ready availability of murine biological assay agents is an additional asset.

## Conclusions

Radiation-damaged oral tissues showed the increased expression of PRRs and their downstream cytokines. We propose that these innate immune molecules contribute to the pathogenesis of OM and a therapeutic strategy to reduce radiation-induced OM with GM-1111 that targets them. GM-1111 is effective in reducing radiation-induced OM in mice and these effects can be exploited without interfering with radiation therapy to induce tumor regression. Future studies in the human patients will provide further validation of therapeutic effects of GM-1111.

## Supporting information

S1 Fig Chemical structure of GM-1111. (TIF)

S2 Fig Photomicrograph of radiation-induced lesion in the dorsal surface of the intermolar eminence in the tongue. The epithelial surface in the tissue sample from the 20 Gy/PBS treatment group. Top panel with 10x original magnification. Boxed regions are further magnified (40x) for better view of microbial colonization (**a**, purple fuzzy stained regions, arrowheads), polymorphonuclear leukocytes (PMNs, arrows in b, c, and d).
(TIF)

S3 Fig Altered gene expression in radiation-damaged lesions. Multiplex gene expression analyses of pattern recognition receptors in the tongue tissues. Tissue samples are from the animals irradiated with either 15 Gy or 20 Gy and treated with PBS. Tongues were harvested 1, 5, and 8 days after the irradiation. Bars represent the mean values and the error bars are SEM. Number of samples: n = 16 for Control (0 Gy/PBS); n = 6 for 15 Gy/PBS and 20 Gy/PBS at day 1, n = 5 for 15 Gy/PBS and n = 6 for 20 Gy/PBS at day 5; n = 6 for 15 Gy/PBS and n = 9 for 20 Gy/PBS at day 8.
(TIF)

S4 Fig Altered gene expression in radiation-damaged lesions. Multiplex gene expression analyses of cytokines in the tongue tissues. Tissue samples are from the animals irradiated with either 15 Gy or 20 Gy and treated with PBS. Tongues were harvested 1, 5, and 8 days after the irradiation. Bars represent the mean values and the error bars are SEM. Number of samples: n = 16 for Control (0 Gy/PBS); n = 6 for 15 Gy/PBS and 20 Gy/PBS at day 1, n = 5 for 15 Gy/PBS and n = 6 for 20 Gy/PBS at day 5; n = 6 for 15 Gy/PBS and n = 9 for 20 Gy/PBS at day 8.
(TIF)
