# Supplementary figures and images for: GM-1111 reduces radiation-induced oral mucositis in mice by targeting pattern recognition receptor-mediated inflammatory signaling
Source: PLoS One. 2021 Mar 26;16(3):e0249343. doi: 10.1371/journal.pone.0249343 (PMC7997003; doi:10.1371/journal.pone.0249343)

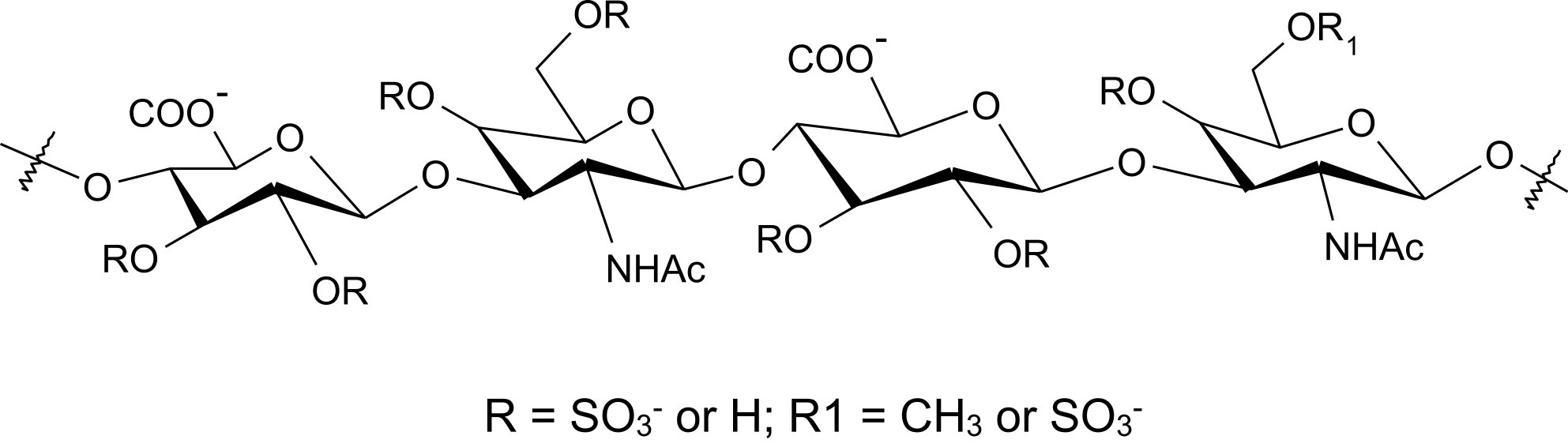

Supplement: S1 Fig — (TIF) [file pone.0249343.s001.tif]

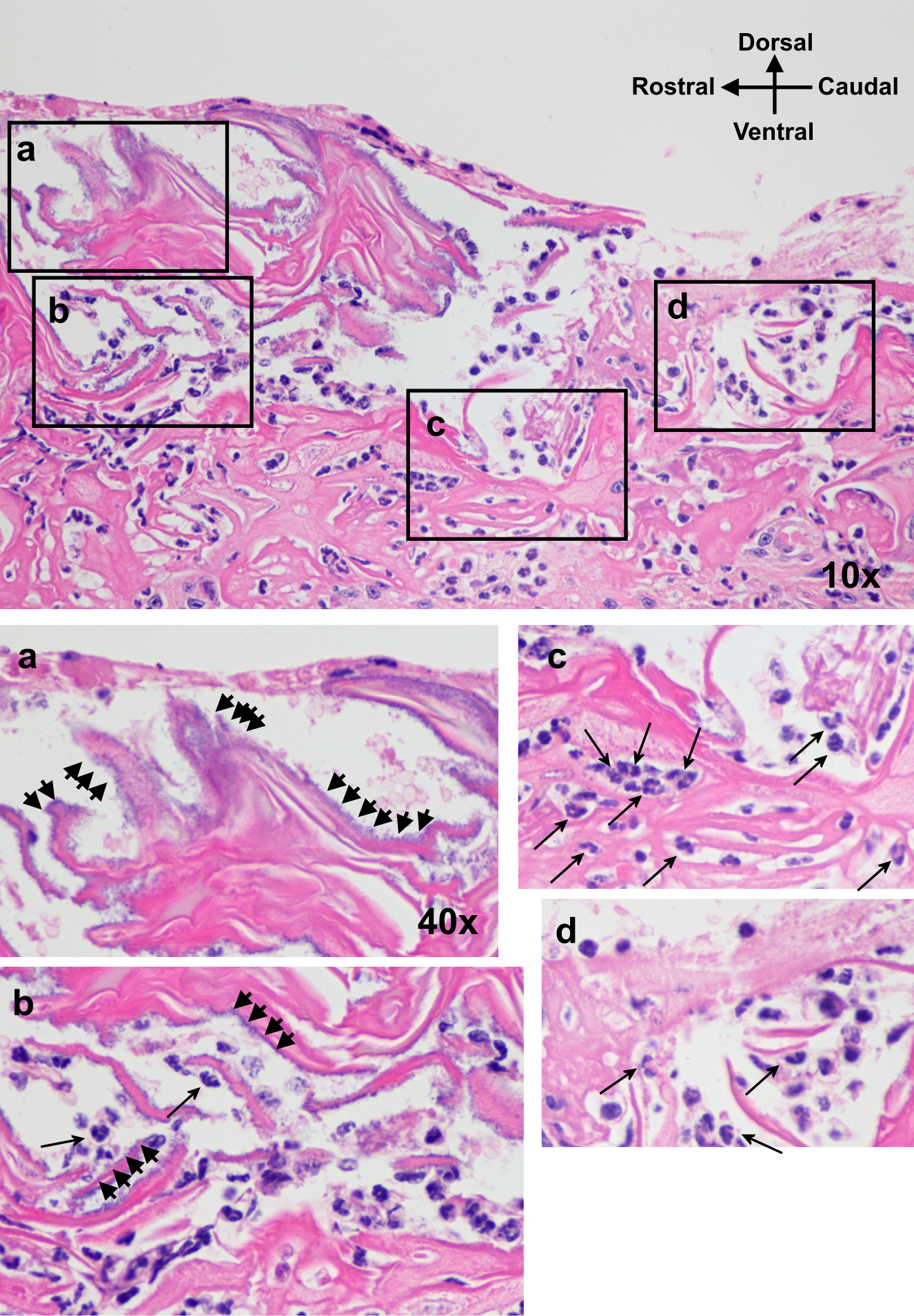

Supplement: S2 Fig — The epithelial surface in the tissue sample from the 20 Gy/PBS treatment group. Top panel with 10x original magnification. Boxed regions are further magnified (40x) for better view of microbial colonization (a, purple fuzzy stained regions, arrowheads), polymorphonuclear leukocytes (PMNs, arrows in b, c, and d). (TIF) [file pone.0249343.s002.tif]

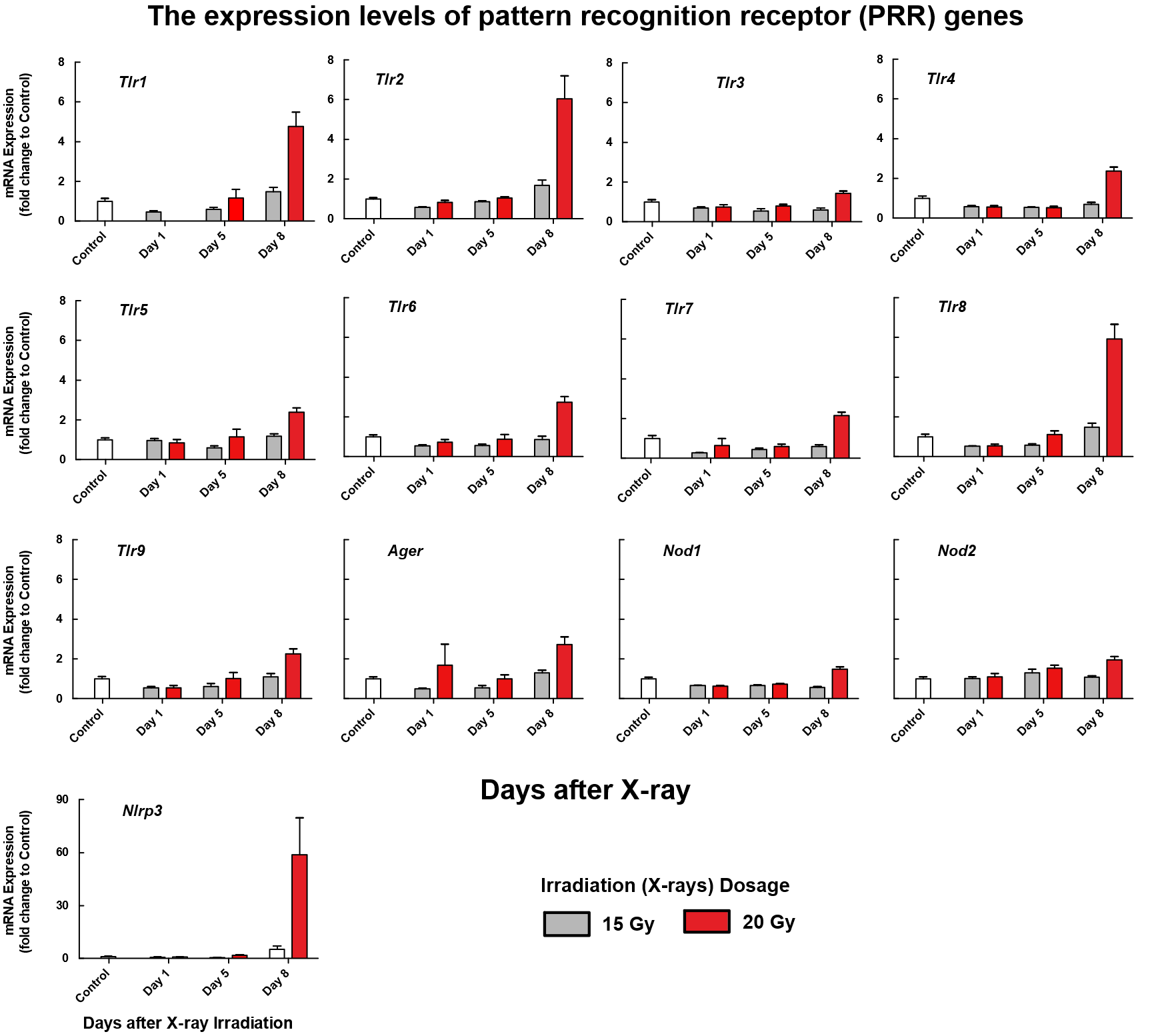

Supplement: S3 Fig — Multiplex gene expression analyses of pattern recognition receptors in the tongue tissues. Tissue samples are from the animals irradiated with either 15 Gy or 20 Gy and treated with PBS. Tongues were harvested 1, 5, and 8 days after the irradiation. Bars represent the mean values and the error bars are SEM. Number of samples: n = 16 for Control (0 Gy/PBS); n = 6 for 15 Gy/PBS and 20 Gy/PBS at day 1, n = 5 for 15 Gy/PBS and n = 6 for 20 Gy/PBS at day 5; n = 6 for 15 Gy/PBS and n = 9 for 20 Gy/PBS at day 8. (TIF) [file pone.0249343.s003.tif]

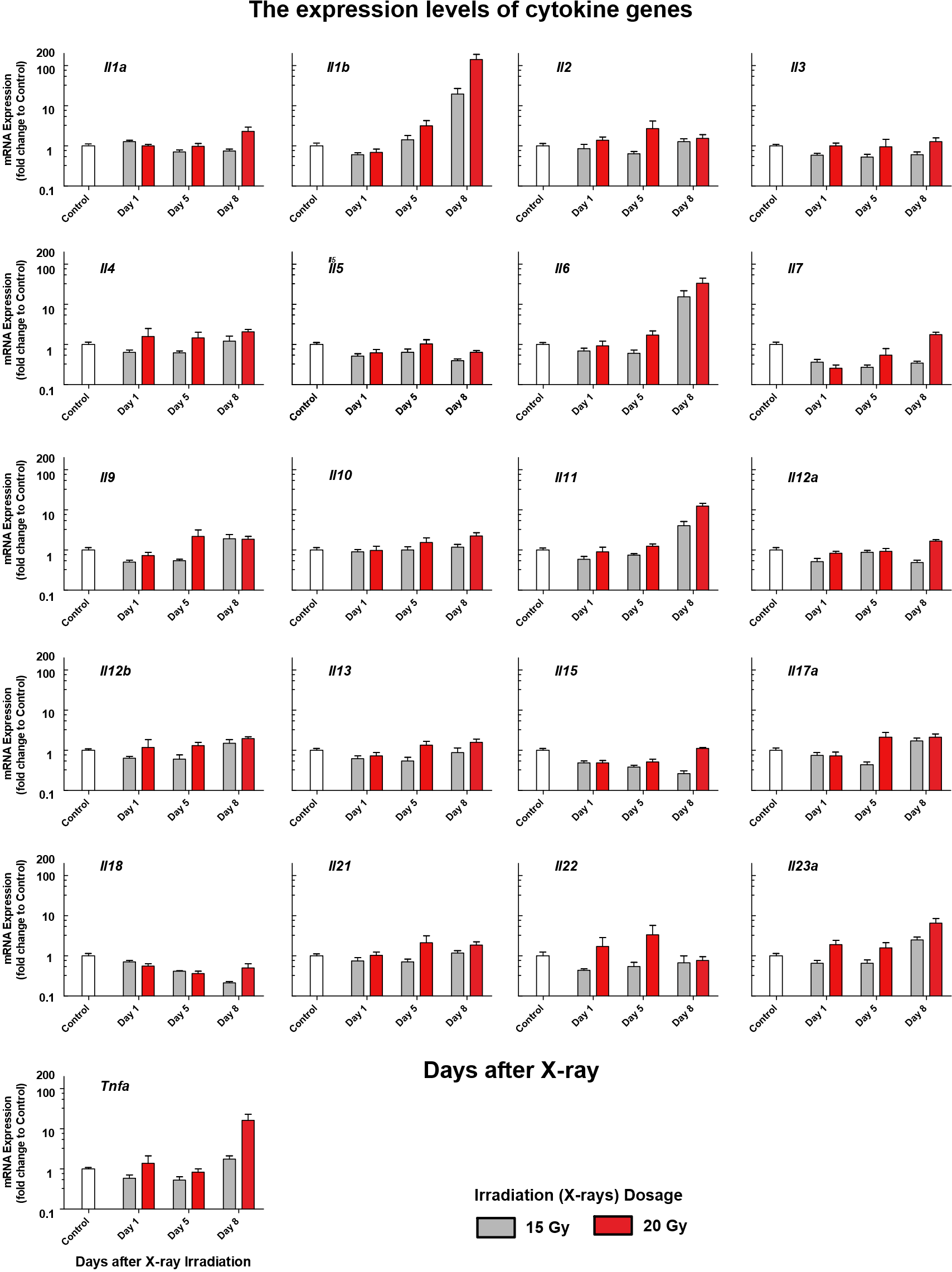

Supplement: S4 Fig — Multiplex gene expression analyses of cytokines in the tongue tissues. Tissue samples are from the animals irradiated with either 15 Gy or 20 Gy and treated with PBS. Tongues were harvested 1, 5, and 8 days after the irradiation. Bars represent the mean values and the error bars are SEM. Number of samples: n = 16 for Control (0 Gy/PBS); n = 6 for 15 Gy/PBS and 20 Gy/PBS at day 1, n = 5 for 15 Gy/PBS and n = 6 for 20 Gy/PBS at day 5; n = 6 for 15 Gy/PBS and n = 9 for 20 Gy/PBS at day 8. (TIF) [file pone.0249343.s004.tif]
